# An Analysis of 3-Year Outcomes Following Canaloplasty for the Treatment of Open-Angle Glaucoma

**DOI:** 10.1155/2017/2904272

**Published:** 2017-09-29

**Authors:** Mahmoud A. Khaimi, Justin D. Dvorak, Kai Ding

**Affiliations:** ^1^Dean McGee Eye Institute, University of Oklahoma Health Sciences Center, Oklahoma City, OK, USA; ^2^Department of Biostatistics and Epidemiology, University of Oklahoma Health Sciences Center, Oklahoma City, OK, USA

## Abstract

**Purpose:**

To report 3-year results investigating the safety and efficacy of canaloplasty for open-angle glaucoma.

**Setting:**

University of Oklahoma, Dean McGee Eye Institute, Oklahoma, United States of America.

**Design:**

Nonrandomized single-center retrospective chart review.

**Methods:**

Adult open-angle glaucoma eyes underwent canaloplasty or combined cataract-canaloplasty surgery. A tensioning suture was placed into Schlemm's canal in all eyes. Primary endpoints included the mean IOP and mean number of glaucoma medications at each follow-up visit. Secondary endpoints included visual acuity and surgical/postsurgical complications.

**Results:**

The study cohort included 277 eyes (mean age, 72.8 years). Overall, the mean baseline IOP of 19.7 mmHg was reduced to 14.3 mmHg,14.0 mmHg, and 15.2 mmHg at 1, 2, and 3 years, respectively (*p* < 0.001). The average medicine use was reduced from 2.1 preoperatively to 0.4 at 12 months, and 0.5 and 0.6 at two and three years, respectively (*p* < 0.001). The frequency of surgical and postsurgical complications was low with no serious adverse events recorded.

**Conclusion:**

Canaloplasty was safe and effective in achieving long-term IOP reductions and reduced dependence on antiglaucoma medications.

## 1. Introduction

Until relatively recently, treatment for primary open-angle glaucoma (POAG) was restricted to medical therapy or traditional glaucoma surgeries such as drainage shunts and trabeculectomy, or laser trabeculoplasty. Trabeculectomy has a well-established IOP-lowering effect; however, it is also associated with numerous immediate and delayed postoperative complications, many of which stem from the creation of a subconjunctival bleb [1–6]. Furthermore, although it is known that suboptimal ocular outflow is a key factor in the development of glaucoma, trabeculectomy works by circumventing, rather than restoring, natural ocular outflow. In contrast, canaloplasty, a modification of viscocanalostomy, lowers IOP both safely and effectively by restoring the physiological outflow pathways [7, 8]. Matlach et al. demonstrated that there was no significant difference in lowering medium IOP with canaloplasty compared to trabeculectomy [9].

Canaloplasty is a nonperforating, blebless technique, eliminating many of the complications frequently seen with trabeculectomy. Even those complications that are associated with the procedure are usually transient and resolve quickly [10]. This is in contrast to trabeculectomy, where bleb-related complications may occur years after the operation, thereby placing a heavy burden of postoperative care on both the patient and the physician [11]. Although canaloplasty is usually indicated for patients with POAG who have not undergone previous filtration surgery, there is evidence to suggest it may be considered in patients for whom other types of surgery have failed [12], and it may also be safely combined with cataract surgery [13].

Prior studies have reported on 1-, 2-, and 3-year results from prospective clinical trials of canaloplasty, which showed significant reductions in IOP and glaucoma medication usage in conjunction with an excellent safety profile [14–17]. The 3-year study results reported herein describe the long-term safety and efficacy of canaloplasty performed by a single surgeon on a large series of patients. Thus, our work intends to verify and add to the long-term outcomes of canaloplasty to further establish it as a reliably efficacious and safe modality.

## 2. Patients and Methods

### 2.1. Design

This paper presents the 3-year results of a single-center retrospective chart review evaluating the intervention of canaloplasty at the University of Oklahoma, Dean McGee Eye Institute, performed by a single surgeon (MK) between January 2010 and December 2012. The cohort included 277 eyes affected by open-angle glaucoma. The study was performed in accordance with the principles stated in the Declaration of Helsinki. The current investigation was designed to demonstrate the safety and efficacy of canaloplasty in reducing intraocular pressure (IOP) and glaucoma medication dependence in the treatment of open-angle glaucoma (OAG). The protocol was approved by the institutional review board (IRB) at the University of Oklahoma.

All patients provided informed consent. All enrollees had a complete baseline ophthalmic examination prior to surgery that included a history of glaucoma, medication use, IOP, best-corrected visual acuity (BCVA), gonioscopy, slit lamp, and fundus examination. Postoperative follow-up examinations were at 1 day, 1 week, and 1, 3, 6, 12, 18, 24, and 36 months. All relevant information was recorded at each follow-up visit, including IOP measurements, BCVA, slit lamp examination, gonioscopy, ophthalmic medications, and adverse events. Primary endpoints included the mean IOP and mean number of glaucoma medications at each follow-up visit. Secondary endpoints included surgical/postsurgical complications. Visual acuity codes such as count fingers (CF), hand motion (HM), and light perception (LP) are converted to LogMAR following Lee et al. [18]. For a given time point, the absence of chart notes indicating hyphema (in the presence of other non-missing data collected from the same visit) was taken to indicate lack of hyphema. If the patient did not complete a visit at the given time point, the data were treated as missing.

### 2.2. Patient Selection

All patients were at a minimum 18 years of age at the time of enrollment, able to provide informed consent, and were scheduled for glaucoma surgery or combined cataract and glaucoma surgery. Inclusion criteria for this study included a glaucoma diagnosis of primary open-angle glaucoma (POAG), pigmentary glaucoma, or exfoliative glaucoma. Patients with advanced glaucoma exhibiting visual field loss were not excluded from the study. Patients with baseline IOP < 16 mmHg were not excluded from the study, and none of the patients had the utilization of antifibrotics. Patients with more than two laser trabeculoplasty (LTP) procedures were excluded. Other exclusion criteria included neovascular disease, uveitis, chronic angle closure glaucoma, angle recession, and developmental or secondary glaucoma. The protocol allowed for previous surgeries that would not interfere with complete circumferential catheterization of Schlemm's canal.

### 2.3. Surgical Technique

Canaloplasty was performed using an iTrack™ microcatheter to circumferentially viscodilate and intubate Schlemm's canal with a tensioning suture, as previously described [19].

### 2.4. Statistical Analysis

The primary endpoints included the IOP and number of antiglaucoma medications at 1 day, 1 week, and 1, 3, 6, 12, 18, 24, and 36 months. The secondary endpoints included visual acuity and surgical/postsurgical complications. Descriptive statistics (mean, SD, count, percent, etc.) were used to summarize the data. Comparisons of IOP, number of medications, and visual acuity were made against baseline at each time point via paired *t*-tests or Wilcoxon's signed-rank tests, as appropriate. Two-tailed *p* values less than 0.05 were considered statistically significant. SAS (version 9.4, SAS Institute) was used for the analysis of the data.

## 3. Results

### 3.1. Demographics

The study cohort consisted of 277 eyes with a mean age of 72.8 ± 10.9 years who met inclusion and exclusion criteria, provided consent for long-term follow-up, and completed baseline visits.  Table 1 shows the patients' demographics. Patients were predominantly white and female, with the majority (92.1%) of the cohort diagnosed with POAG.

### 3.2. Change in Intraocular Pressure and Antiglaucoma Medication Use

Table 2 shows the efficacy results for all enrolled eyes in terms of IOP reduction and the number of glaucoma medications used. The IOP was significantly decreased at all time points compared to baseline (all *p* < 0.001). Similarly, the medication use was significantly decreased at all postoperative time points compared to baseline (*p* < 0.001). For all eyes, the mean baseline IOP of 19.7 ± 6.7 mmHg was reduced to 14.3 ± 4.6 mmHg at 12 months, 14.0 ± 4.2 mmHg at 24 months, and 15.2 ± 4.3 mmHg at 36 months (*p* < 0.001). The mean medicine use was reduced from 2.1 ± 1.2 before surgery to 0.4 ± 0.8 at 12 months, 0.5 ± 0.9 at 24 months, and 0.6 ± 0.9 at 36 months (*p* < 0.001) (Table 2).

For the 150 eyes which underwent canaloplasty alone without phacoemulsification, the mean IOP reduced from 21.1 ± 7.2 mmHg at baseline to 14.2 ± 4.6 mmHg at 12 months, 13.3 ± 4.1 mmHg at 24 months and 15.0 ± 4.6 mmHg at 36 months (*p* < 0.001) (Table 3). Medication use for the same subset was reduced from 2.2 ± 1.3 at baseline to 0.5 ± 0.9 at 12 months, 0.5 ± 0.8 at 24 months, and 0.5 ± 0.8 at 36 months (*p* < 0.001) (Table 3).

A statistically significant reduction in IOP was also observed in the 127 eyes undergoing phacocanaloplasty from a mean of 18.1 ± 5.6 mmHg at baseline to 14.4 ± 4.6 mmHg, 14.7 ± 4.4 mmHg, and 15.4 ± 4.0 mmHg at 12, 24, and 36 months, respectively (*p* < 0.001 for 12 and 24 months, *p* = 0.002 for 36 months) (Table 4). Mean medication use was reduced from 2.0 ± 1.1 at baseline to 0.4 ± 0.7 at 12 months, 0.6 ± 0.9 at 24 months, and 0.7 ± 1.1 at 36 months (*p* < 0.001).

Table 5 shows the complete and qualified success rate for canaloplasty broken down by IOP level at 6 months, 12 months, 24 months, and 36 months after surgery. Using the criteria of IOP ≤ 21 mmHg, complete success (defined as no use of antiglaucoma medication) was attained in 82.5% of patients at 6 months, 67.4% at 24 months, and 57.8% at 36 months. For IOP ≤ 15 mmHg and ≥25% IOP reduction compared to baseline, complete success was deemed to be achieved in 34.0% at 6 months, 36.2% at 24 months, and 26.6% at 36 months. Qualified success, defined as patients using two or fewer antiglaucoma medications and an IOP ≤ 21mmHG, was achieved in 91.8% of patients at 6 months, 92.2% at 24 months, and 87.5% at 36 months. Similar complete and qualified success rates were also observed for eyes with canaloplasty only (no phacocanaloplasty) (Table 6) and also for the subset of eyes with phacocanaloplasty only (Table 7) at the specified follow-up time points.

### 3.3. Intraoperative and Postoperative Complications

#### 3.3.1. Hyphema

The percentage of eyes with hyphema at a given time point is presented in Table 8. Except at day 1, where about half of the eyes had hyphema, the rate of this complication was low at later visits. Hyphema also tended to resolve quickly. Of the 144 eyes (52.8%) with hyphema at 1-day post-op, 99 (68.8%) were confirmed resolved by week 1, 139 (96.5%) by month 1, and 125 (86.8%) by month 3. The drop-off in resolved hyphema at month 3 is likely due to loss to follow-up.

### 3.4. Visual Recovery

For all eyes, mean pre-op visual acuity (LogMAR) was 0.31 ± 0.35 with a mean Snellen fraction of 20/41.1. Visual acuities were found to be significantly worse at 1 day, 1 week, and 1 month following the procedure. Post-op visual acuity was found to be 0.85 ± 0.53 with a Snellen fraction of 20/140.2 (*p* < 0.0001) at 1 day, 0.57 ± 0.42 with a Snellen fraction of 20/74.8 (*p* < 0.0001) at 1 week, and 0.40 ± 0.35 with a Snellen fraction of 20/49.8 (*p* < 0.0001) at 1 month (Table 2). The post-op visual acuity was significantly better starting at 12 months, where 185 eyes had a visual acuity of 0.24 ± 0.40 with a Snellen fraction of 20/34.8 (*p* < 0.05). At 18 months, 172 eyes had a visual acuity of 0.23 ± 0.36 with a Snellen fraction of 20/33.8 (*p* = 0.06). Two years after surgery, 143 eyes had a visual acuity of 0.22 ± 0.29 with a Snellen fraction of 20/33.3 (*p* < 0.001). At 3 years, visual acuity was not significantly better or worse, found to be 0.20 ± 0.24 with a Snellen fraction of 20/31.5 (*p* = 0.29) among 65 eyes (Table 2).

Mean and SD for visual acuity (LogMAR) are presented in Table 3 for eyes undergoing canaloplasty alone (*n* = 150) and in Table 4 for eyes undergoing phacocanaloplasty (*n* = 127), along with corresponding means in Snellen fractions and paired *t*-test results (versus pre-op VA in LogMAR). Rows in bold represent significantly worse visual acuity compared to pre-op, whereas rows in italic represent improved visual acuity. Broadly speaking, visual acuity worsens significantly after surgery but may return to preoperative values by around 3–12 months postoperatively.

## 4. Discussion

IOP reduction is the mainstay of glaucoma treatment. The 3-year results reported here showed significant and sustained pressure lowering accompanied by a low incidence of postoperative complications. The reductions in IOP achieved over the long-term follow-up period are in line with previously reported studies in the literature and notably the landmark multicenter prospective trial carried out at 15 clinical sites in the United States, Great Britain, and Germany in 2005 [17].

This groundbreaking study of Lewis et al. included 157 eyes of 157 OAG patients with a historical pressure of 21 mmHg or higher, with many of them on maximum tolerated medical therapy. Canaloplasty procedures were carried out on 121 eyes while 36 eyes underwent phacocanaloplasty. Of the 89 procedures performed with a successful placement of a suture, there was a 34% mean decrease in IOP from baseline (23.5 ± 4.5 to 15.5 ± 3.5 mmHg) and a 53% mean reduction in postoperative medications (1.9 ± 0.8 to 0.9 ± 0.9) at three-year follow-up. When phacoemulsification was combined with canaloplasty and successful suture placement, 27 eyes had a 42% mean decrease in IOP (23.5 ± 5.2 to 13.6 ± 3.6 mmHg) and an 80% mean reduction of postoperative medications (1.5 ± 1 to 0.3 ± 0.5).

Another long-term study by Bull et al. investigated the efficacy of canoloplasty and phacocanaloplasty on European eyes with open-angle glaucoma [7]. One hundred and nine eyes were included in the study with successful intracanalicular suture tensioning occurring in 98 eyes (89.9%), canaloplasty alone performed in 93 eyes (85.3%) and phacocanaloplasty performed in 16 eyes (14.7%) with 3-year follow-up data available for 96 eyes (88.1%). In the canaloplasty alone group, the mean baseline IOP of 23.0 ± 4.3 mmHg on 1.9 ± 0.7 medications was significantly reduced to 15.1 ± 3.1 mmHg on 0.9 ± 0.9 medications while the phacocanaloplasty group's mean baseline IOP of 24.3 ± 6.0 mmHg on 1.5 ± 1.2 medications was significantly reduced to 13.8 ± 3.2 mmHg on 0.5 ± 0.7 medications (*p* < 0.0001). The outcomes in several recently published studies have equaled or surpassed those of the 2005 trial. In 2011, Grieshaber et al. published the results of a prospective study of 32 patients with OAG in which the mean IOP fell from 27.3 ± 5.6 mmHg preoperatively to 12.8 ± 1.5 mmHg at 12 months and 13.1 ± 1.2 mmHg at 18 months [16]. A more recent study by Brusini of 214 eyes from 185 OAG patients with a maximum of four-year follow-up reported a mean IOP reduction of 42.2% [20].

Compared to these previous trials, our large-scale study of 277 eyes with successful suture tensioning demonstrated a combined 23% reduction in IOP from baseline (19.7 ± 6.7 to 15.2 ± 4.3 mmHg) and 71% reduction in medication use (2.1 ± 1.2 to 0.6 ± 0.9) at three-year follow-up. The 150 patients undergoing canaloplasty alone had a 29% reduction in IOP (21.1 ± 7.2 to 15.0 ± 4.6 mmHg) and 77% reduction in medication use postoperatively (2.2 ± 1.3 to 0.5 ± 0.8) at three years. The 127 eyes undergoing phacocanaloplasty had a 17% mean decrease in IOP (18.1 ± 5.6 to 15.1 ± 4.0 mmHg) and a 65% reduction in medication use postoperatively (2.0 ± 1.1 to 0.7 ± 1.1) at three years. These patients had a lower baseline preoperative IOP as they were predominately undergoing surgical intervention for cataract, rather than increased pressure. In all cases, our study reached a similar endpoint when compared to previous studies, with a significant reduction in both IOP and postoperative medication use. This large data set, with 277 procedures performed by a single surgeon, acts to further verify and add to the efficacy of canaloplasty as a viable option for the treatment of OAG. A recent review by Cagini et al. summarizes the potential pitfalls associated with canaloplasty, noting that the meta-analysis of the literature demonstrates a relatively low rate of complications, particularly severe ones, compared to trabeculectomy [21]. Potential intraoperative complications associated with canaloplasty include inability to cannulate Schlemm's canal, Descemet membrane detachment, and improper microcatheter passage [7, 17, 18]. In the current investigation, patients were excluded due to inability to place a suture tension. The most frequent postoperative complications associated with canaloplasty include hyphema or microhyphema, cataract formation, IOP spikes, and hypotony [14]. While transient hyphema was the most common side effect in the current study, a study by Grieshaber et al. has shown that hyphema can, in fact, be considered to be a sign of successful reconnection with the ocular venous system and therefore of good prognosis [22]. Sustained hypotony and related complications, however, did not occur. Our own study confirmed the robust safety profile of canaloplasty and replicated the low rate of intraoperative and postoperative complications reported in previous studies. The high rate of attrition at year three in this study is attributable to few postoperative complications, allowing for prompt referral back to their primary doctors.

## 5. Conclusion

This single-center clinical trial provides further evidence of the significant IOP lowering efficacy of canaloplasty with suture tensioning, with continued control through a 3-year postoperative period. The risk profile of canaloplasty was favorable and consistent with the well-documented, lower risks associated with other nonpenetrating procedures. Canaloplasty's safety profile and long-term efficacy make it a viable option for the majority of glaucoma patient types. It can be used in conjunction with existing drug-based glaucoma treatments, after laser or other types of incisional surgery, and does not preclude or affect the outcome of future surgery. It is a procedure that offers a high probability of success in effectively lowering IOP both alone and combined with phacoemulsification.

## Figures and Tables

**Table 1:** Study group demographics of all eyes.

Variable	Descriptive summary
Total eyes	277
Age (years)	
Mean ± SD	72.8 ± 10.9
Range	40–100
Sex: count (%)	
Female	151 (54.5)
Male	126 (45.5)
Race: count (%)	
Asian	1 (0.4)
African American	39 (15.0)
Caucasian	188 (72.3)
Hispanic	7 (2.7)
Native American	25 (9.6)
Missing	17
Eye treated: count (%)	
Right eye	142 (51.3)
Left eye	135 (48.7)
Combined cataract procedure: count (%)	
Yes (phacocanaloplasty)	127 (45.8)
No (canaloplasty only)	150 (54.2)

**Table 2:** Outcome measurements by visit (all eyes).

Visit	IOP (mmHg)			Meds (number)			Visual acuity^1^			
	Mean ± SD	*N*	*p* value	Mean ± SD	*N*	*p* value	Mean ± SD	Snellen	*N*	*p* value
Pre-op	19.7 ± 6.7	277		2.1 ± 1.2	277		0.31 ± 0.35	20/41.1	277	
Day 1	10.2 ± 6.0	271	<0.001	0.0 ± 0.1	277	<0.001	**0.85 ± 0.53**	**20/140.2**	**272**	**<0.0001**
Wk 1	15.3 ± 7.1	271	<0.001	0.0 ± 0.2	276	<0.001	**0.57 ± 0.42**	**20/74.8**	**276**	**<0.0001**
Mo 1	13.9 ± 6.1	267	<0.001	0.1 ± 0.4	274	<0.001	**0.40 ± 0.35**	**20/49.8**	**275**	**<0.0001**
Mo 3	14.4 ± 5.4	234	<0.001	0.1 ± 0.5	228	<0.001	0.29 ± 0.34	20/39.4	239	0.47
Mo 6	14.6 ± 5.1	198	<0.001	0.2 ± 0.6	205	<0.001	0.25 ± 0.37	20/35.4	202	0.07
Mo 12	14.3 ± 4.6	181	<0.001	0.4 ± 0.8	186	<0.001	*0.24 ± 0.40*	*20/34.8*	*185*	*0.04*
Mo 18	15.0 ± 4.6	167	<0.001	0.6 ± 0.8	144	<0.001	0.23 ± 0.36	20/33.8	172	0.06
Yr 2	14.0 ± 4.2	144	<0.001	0.5 ± 0.9	159	<0.001	*0.22 ± 0.29*	*20/33.3*	*143*	*<0.001*
Yr 3	15.2 ± 4.3	66	<0.001	0.6 ± 0.9	74	<0.001	0.20 ± 0.24	20/31.5	65	0.29

^1^LogMAR unless indicated.

**Table 3:** Outcome measurements by visit (eyes with canaloplasty only).

Visit	IOP (mmHg)			Meds (number)			Visual acuity^1^			
	Mean ± SD	*N*	*p* value	Mean ± SD	*N*	*p* value	Mean ± SD	Snellen	*N*	*p* value
Pre-op	21.1 ± 7.2	150		2.2 ± 1.3	150		0.23 ± 0.33	20/34.1	150	
Day 1	8.0 ± 5.0	149	<0.001	0.0 ± 0.1	150	<0.001	**0.79 ± 0.50**	**20/124.6**	**148**	**<0.0001**
Wk 1	15.3 ± 7.5	146	<0.001	0.0 ± 0.1	149	<0.001	**0.55 ± 0.43**	**20/71.7**	**149**	**<0.0001**
Mo 1	15.5 ± 5.6	145	<0.001	0.0 ± 0.3	148	<0.001	**0.40 ± 0.39**	**20/50.2**	**149**	**<0.0001**
Mo 3	14.8 ± 5.1	127	<0.001	0.1 ± 0.6	123	<0.001	**0.31 ± 0.39**	**20/40.7**	**131**	**<0.001**
Mo 6	14.9 ± 5.4	105	<0.001	0.2 ± 0.6	109	<0.001	**0.28 ± 0.44**	**20/38.1**	**107**	**0.03**
Mo 12	14.2 ± 4.6	102	<0.001	0.5 ± 0.9	102	<0.001	0.28 ± 0.48	20/37.7	101	0.17
Mo 18	14.6 ± 4.5	89	<0.001	0.5 ± 0.8	76	<0.001	**0.28 ± 0.44**	**20/38.0**	**89**	**0.04**
Yr 2	13.3 ± 4.1	75	<0.001	0.5 ± 0.8	85	<0.001	0.26 ± 0.36	20/36.7	74	0.20
Yr 3	15.0 ± 4.6	39	<0.001	0.5 ± 0.8	43	<0.001	*0.16 ± 0.18*	*20/29.2*	*38*	*0.05*

^1^LogMAR unless indicated.

**Table 4:** Outcome measurements by visit (eyes with phacocanaloplasty).

Visit	IOP (mmHg)			Meds (number)			Visual acuity^1^			
	Mean ± SD	*N*	*p* value	Mean ± SD	*N*	*p* value	Mean ± SD	Snellen	*N*	*p* value
Pre-op	18.1 ± 5.6	127		2.0 ± 1.1	127		0.41 ± 0.35	20/51.3	127	
Day 1	12.9 ± 5.9	122	<0.001	0.0 ± 0.2	127	<0.001	**0.91 ± 0.56**	**20/161.4**	**124**	**<0.0001**
Wk 1	15.3 ± 6.6	125	<0.001	0.0 ± 0.3	127	<0.001	**0.59 ± 0.40**	**20/78.5**	**127**	**<0.0001**
Mo 1	12.0 ± 6.1	122	<0.001	0.1 ± 0.5	126	<0.001	0.39 ± 0.29	20/49.4	126	0.72
Mo 3	13.8 ± 5.7	107	<0.001	0.1 ± 0.4	105	<0.001	*0.28 ± 0.28*	*20/37.8*	*108*	*<0.001*
Mo 6	14.4 ± 4.6	93	<0.001	0.2 ± 0.6	96	<0.001	*0.21 ± 0.28*	*20/32.6*	*95*	*<0.0001*
Mo 12	14.4 ± 4.6	79	<0.001	0.4 ± 0.7	84	<0.001	*0.20 ± 0.27*	*20/31.5*	*84*	*<0.0001*
Mo 18	15.5 ± 4.8	78	<0.001	0.6 ± 0.9	68	<0.001	*0.17 ± 0.22*	*20/29.9*	*83*	*<0.0001*
Yr 2	14.7 ± 4.4	69	<0.001	0.6 ± 0.9	74	<0.001	*0.18 ± 0.17*	*20/30.1*	*69*	*<0.0001*
Yr 3	15.4 ± 4.0	27	0.002	0.7 ± 1.1	31	<0.001	*0.24 ± 0.29*	*20/35.1*	*27*	*0.03*

^1^LogMAR unless indicated.

**Table 5:** Success rates (all eyes).

Success type	6 months (*N* = 194)	12 months (*N* = 176)	Year 2 (*N* = 141)	Year 3 (*N* = 64)
Complete success^1^				
≤21 mmHg	82.5	69.3	67.4	57.8
≤21 mmHg and ≥25% IOP reduction^3^	40.7	44.9	40.4	28.1
≤18 mmHg	72.2	63.6	59.6	53.1
≤18 mmHg and ≥25% IOP reduction^3^	39.2	44.3	38.3	28.1
≤15 mmHg	53.6	51.1	48.9	46.9
≤15 mmHg and ≥25% IOP reduction^3^	34.0	36.9	36.2	26.6
Qualified success^2^				
≤21 mmHg	91.8	93.2	92.2	87.5
≤21 mmHg and ≥25% IOP reduction^3^	44.3	54.0	56.7	35.9
≤18 mmHg	78.9	81.3	82.3	75.0
≤18 mmHg and ≥25% IOP reduction^3^	42.3	51.7	53.9	34.4
≤15 mmHg	57.7	63.1	68.1	64.1
≤15 mmHg and ≥25% IOP reduction^3^	36.6	43.8	51.1	32.8

^1^No use of antiglaucoma medication. ^2^Use of ≤2 (including 0) antiglaucoma medications. ^3^IOP reduction from baseline/pre-op measurement.

**Table 6:** Success rates (eyes with canaloplasty only).

Success type	6 months (*N* = 103)	12 months (*N* = 100)	Year 2 (*N* = 74)	Year 3 (*N* = 37)
Complete success^1^				
≤21 mmHg	83.5	70	70.3	64.9
≤21 mmHg and ≥25% IOP reduction^3^	43.7	48	48.6	43.2
≤18 mmHg	70.9	64	62.2	59.5
≤18 mmHg and ≥25% IOP reduction^3^	41.7	47	47.3	43.2
≤15 mmHg	52.4	51	54.1	56.8
≤15 mmHg and ≥25% IOP reduction^3^	36.9	40	44.6	40.5
Qualified success^2^				
≤21 mmHg	92.2	93	95.9	94.6
≤21 mmHg and ≥25% IOP reduction^3^	48.5	57	68.9	48.6
≤18 mmHg	77.7	80	83.8	78.4
≤18 mmHg and ≥25% IOP reduction^3^	45.6	54	66.2	45.9
≤15 mmHg	56.3	62	74.3	67.6
≤15 mmHg and ≥25% IOP reduction^3^	39.8	46	63.5	43.2

^1^No use of antiglaucoma medication. ^2^Use of ≤2 (including 0) antiglaucoma medications. ^3^IOP reduction from baseline/pre-op measurement.

**Table 7:** Success rates (eyes with phacocanaloplasty).

Success type	6 months (*N* = 91)	12 months (*N* = 76)	Year 2 (*N* = 67)	Year 3 (*N* = 27)
Complete success^1^				
≤21 mmHg	81.3	68.4	64.2	48.1
≤21 mmHg and ≥25% IOP reduction^3^	37.4	40.8	31.3	7.4
≤18 mmHg	73.6	63.2	56.7	44.4
≤18 mmHg and ≥25% IOP reduction^3^	36.3	40.8	28.4	7.4
≤15 mmHg	54.9	51.3	43.3	33.3
≤15 mmHg and ≥25% IOP reduction^3^	30.8	32.9	26.9	7.4
Qualified success^2^				
≤21 mmHg	91.2	93.4	88.1	77.8
≤21 mmHg and ≥25% IOP reduction^3^	39.6	50.0	43.3	18.5
≤18 mmHg	80.2	82.9	80.6	70.4
≤18 mmHg and ≥25% IOP reduction^3^	38.5	48.7	40.3	18.5
≤15 mmHg	59.3	64.5	61.2	59.3
≤15 mmHg and ≥25% IOP reduction^3^	33.0	40.8	37.3	18.5

^1^No use of antiglaucoma medication. ^2^Use of ≤2 (including 0) antiglaucoma medications. ^3^IOP reduction from baseline/pre-op measurement.

**Table 8:** Presence of hyphema by visit (all eyes).

Visit	*N*	Present	Absent	% present	Missing
Pre-op	277	0	277	0.0	0
Day 1	273	144	129	52.8	4
Wk 1	274	46	228	16.8	3
Mo 1	267	3	264	1.1	10
Mo 3	234	0	234	0.0	43
